# Chronic Diseases and How to Manage Them

**Published:** 1894-06

**Authors:** Arthur G. Allan

**Affiliations:** New York


### CHRONIC DISEASES AND HOW TO MANAGE THEM.

ARTHUR G. ALLAN, M. D., NEW YORK.

Not long after Hahnemann had discovered and put in practice the law of therapeutics, "*similia similibus curantur*," he noticed that although the law of therapeutics was infallible and of universal application, that there were certain phases of disease that presented difficulties in the way of a cure that made him fearful of the ultimate successful application of the law after all. This set him to work observing cases, and trying to discover, if possible, the reason why remedies that appeared to be strictly indicated failed to produce a permanent and a radical cure. He observed that such conditions as periodical headaches, inflammation of the eyes in children, the so-called bilious attacks, and many other like complaints, though they were promptly relieved by such remedies as Acon., Bell., Bry., Cham., Nux, etc., were not cured, but that these attacks continued to recur, and that the remedies employed acted less promptly and efficiently in succeeding attacks, until finally, though seemingly as well indicated as in the first attack, they absolutely failed to act at all. This led him to the conclusion that he had not the entire case presented in these acute attacks. He observed patients who so suffered, in the interval of comparative health, and found that, though they were free from acute suffering, still they had a variety of symptoms which indicated that they were sick, and that the headache, the sore eyes, or the bilious attack was but a mere fragment of a disease pervading the whole organism, whose extent and nature could not at all be presented by these sudden acute outbursts. It was in this way that Hahnemann discovered and developed his doctrine of psora, and it is to assist the student as well as the practitioner to know how to apply successfully his remedies for the cure of psora that I have presented this paper. It is often not so difficult to select the appropriate antipsoric remedy, but questions often arise as to what should be done under such or such circumstances. To decide this it is often necessary to look through the work on *Chronic Diseases*, or some other work (which, on account of being out of print, one may not possess); so to aid us in such emergencies, I have condensed the most important points regarding chronic diseases into as small a space as possible, in the hope that it will help others as it has often myself.

The phenomena which appear to constitute the disease in an individual case should not be regarded as the whole extent of the chronic disease. It is but a mere fragment of a deep-seated evil, the extent of which may be observed from the new symptoms which appear from time to time, together with the symptoms which the patient presents in the interval of comparative health observed between the acute attacks.

Groups of symptoms of this sort ought not to be treated as separate diseases, but the image of the entire disease must be derived from all the different symptoms together, thus characterizing the disease as a whole.

Evidently, the primitive disease owed its existence to a chronic miasm, which tends to grow worse and never to yield even to the strongest constitution, regular life, or strict diet. This disease is generally the result of a suppressed skin eruption.

Infection, in both acute and chronic diseases, takes place in a moment. Cauterization, washing, burning, excision, etc., are consequently of no avail. The internal disease must be fully developed in the organism before the eruption can appear upon the skin. Syphilis and sycosis can infect the system only by an abraded surface. Psora, on the contrary, taints the system (especially of children) by simply touching the sound skin. The whole organism must first become adapted to the chronic miasm before the eruption can appear on the skin and the disease become fully developed. The time that elapses between the moment when the infection takes place and when the eruption appears is called the period of incubation. Unlike syphilis and sycosis, the secondary symptoms of psora are not communicable.

The action of the antipsoric remedy must not be disturbed by the administration of a remedy for a slight headache, sore throat, diarrhœa, a little pain, colic, etc.

In case the antipsoric remedy brings out symptoms that existed days, weeks, or months previously, they should be left without prescribing a remedy for them.

If the antipsoric remedy brings out symptoms that never existed before and which may be supposed to be inherent in the remedy, then the remedy should be allowed to act for a while undisturbed. Generally such symptoms disappear of themselves; but if they do not, and remain and become troublesome, then they show that the remedy was not correctly chosen. Such symptoms need an antidote, and the antidote to the antipsoric which was administered should be given; but if such an antidote is not known then another antipsoric should be given according to the totality of the existing symptoms.

If, after the administration of an antipsoric remedy, the previously existing symptoms are intensified it should give no cause for uneasiness, as this is the so-called homœopathic aggravation and will gradually disappear.

But if these symptoms should not decrease it shows that the magnitude of the dose was too great and has rendered the cure (reaction) impossible. In such a case an antidote should be given, or, if one is not known, another antipsoric remedy, chosen according to the totality of the symptoms present, should be administered.

In the treatment of psora external applications of any sort are dangerous and should not be employed.

After psora has been suppressed Sulphur alone is never sufficient to cure the case.

With the exception of the itch eruption itself psoric manifestations cannot be cured by one antipsoric remedy, but they require several remedies.

Diet must be regulated; drinks must be regulated.

The use of domestic medicines, perfumes, toothwashes, condiments, spices, mineral waters, mineral baths, and baths either hot or cold must be prohibited.

Moderate exercise should be taken.

Change of locality, as from damp or marshy districts to dry ones.

Religious or philosophical reasoning should be prohibited.

Doses cannot be too small; don't use the remedy improperly; let the remedy act a sufficient length of time undisturbed.

Medicines must be chosen homœopathically and not *ab usu in morbis.*

There is no definite duration of the action of a remedy.

A remedy should be repeated or a new one should be administered in chronic disease only when the old symptoms which had disappeared for a short time show a tendency to return either in the same or a different form.

The duration of action of a remedy is always proportionate to the chronic character of the disease, being shorter as the disease is more acute, and longer as the disease is chronic.

In all cases a remedy must be allowed to act as long as improvement continues.

If the antipsoric remedy produces symptoms that are not homogeneous, and if the mind of the patient becomes depressed, a second dose of the same remedy may become very prejudicial to the patient.

If a remedy when given in a chronic disease produces a sudden and great improvement, there is danger that it has acted merely as a palliative, and the dose should never be repeated.

When improvement ceases, a second dose may be given, but in order to get a stronger action it is best to give the remedy in a different potency.

Except Hepar, Sepia, and Sulphur the antipsoric remedies seldom admit of a favorable repetition.

By frequently changing potencies, changing remedies, or frequently repeating the doses, the system becomes so irritated that remedies lose all their control over it. Under such circumstances hypnotism is valuable to calm the irritated nervous system.

If the most troublesome symptoms of a chronic disease disappear speedily and as if by magic it is no proof that the homœopathic remedy has been properly selected; but this apparent success is deceptive, and shows the medicine to be a mere palliative, and that the original disease will soon reappear. In such a case an antidote to the remedy that has been administered should be given or another antipsoric remedy administered.

Some remedies produce effects and alternating effects. In such cases a second dose of the remedy may be given which will by its antipathic effects produce a permanent improvement.

When a patient suffering from a chronic disease is attacked by an intercurrent disease it interrupts the antipsoric treatment. The antipsoric treatment should be suspended as long as this disease lasts, and should not be resumed until the chronic disease has again commenced to run its course.

If the symptoms of the chronic disease have been modified by the acute disease, the antipsoric remedy must be prescribed strictly according to the existing symptoms without regard to the former remedy which had been prescribed before the attack from the intercurrent disease.

If a patient does not recover promptly and properly from an acute epidemic or sporadic disease it shows that there exists in the organism a psoric miasm for which an antipsoric remedy must be prescribed, even though the acute disease subsided rapidly from the action of the properly selected homœopathic remedy.

The obstinate character of endemic diseases is due to a complication with psora. Man can endure extremes of heat and cold; but the exhalations from marshes and damp regions rouse the latent psora in the organism and develops all sorts of disorders.

In the treatment of chronic diseases—old symptoms—that is, those that have existed the longest disappear the last. "Symptoms disappear in the reverse order of their coming."

Hysteria, epilepsy, etc., may be temporarily improved by an antipsoric remedy, but they can be permanently removed by treatment only when the psoric miasm has been driven from the organism.

When the cure of a chronic disease is about half complete the psoric miasm tends to become latent, but as long as there remains the least symptom it must be eradicated by a remedy; because, if it is left to itself, it will, little by little, develop anew until it again conquers the organism.

It is impossible to estimate the time required for the cure of a chronic disease.

The best time to take an antipsoric remedy is before breakfast and upon an empty stomach. The patient should then remain quiet, without sleeping, and free from mental emotions or mental labor.

An antipsoric remedy should never be taken immediately before or during the menses. Pregnancy offers no obstacles to an antipsoric treatment; on the contrary, an antipsoric remedy may be more needed during pregnancy than at any other time.

As antipsorics are slow in acting, it is sometimes necessary to give a patient an injection to empty the bowel, *but it should contain nothing besides pure water.*

During the antipsoric treatment, baths, either cold or hot, should not be permitted.

